# Supplementary material for: Comprehensive Annotation and Functional Exploration of MicroRNAs in Lettuce
Source: Front Plant Sci. 2021 Dec 24;12:781836. doi: 10.3389/fpls.2021.781836 (PMC8739914; doi:10.3389/fpls.2021.781836)
Supplement: Supplementary file 2 [file Data_Sheet_8.ZIP › GSM2230749.plot/Lsa-miR166h_Lsat_1_v5_gn_4_100080.1_1067_TPlot.pdf]

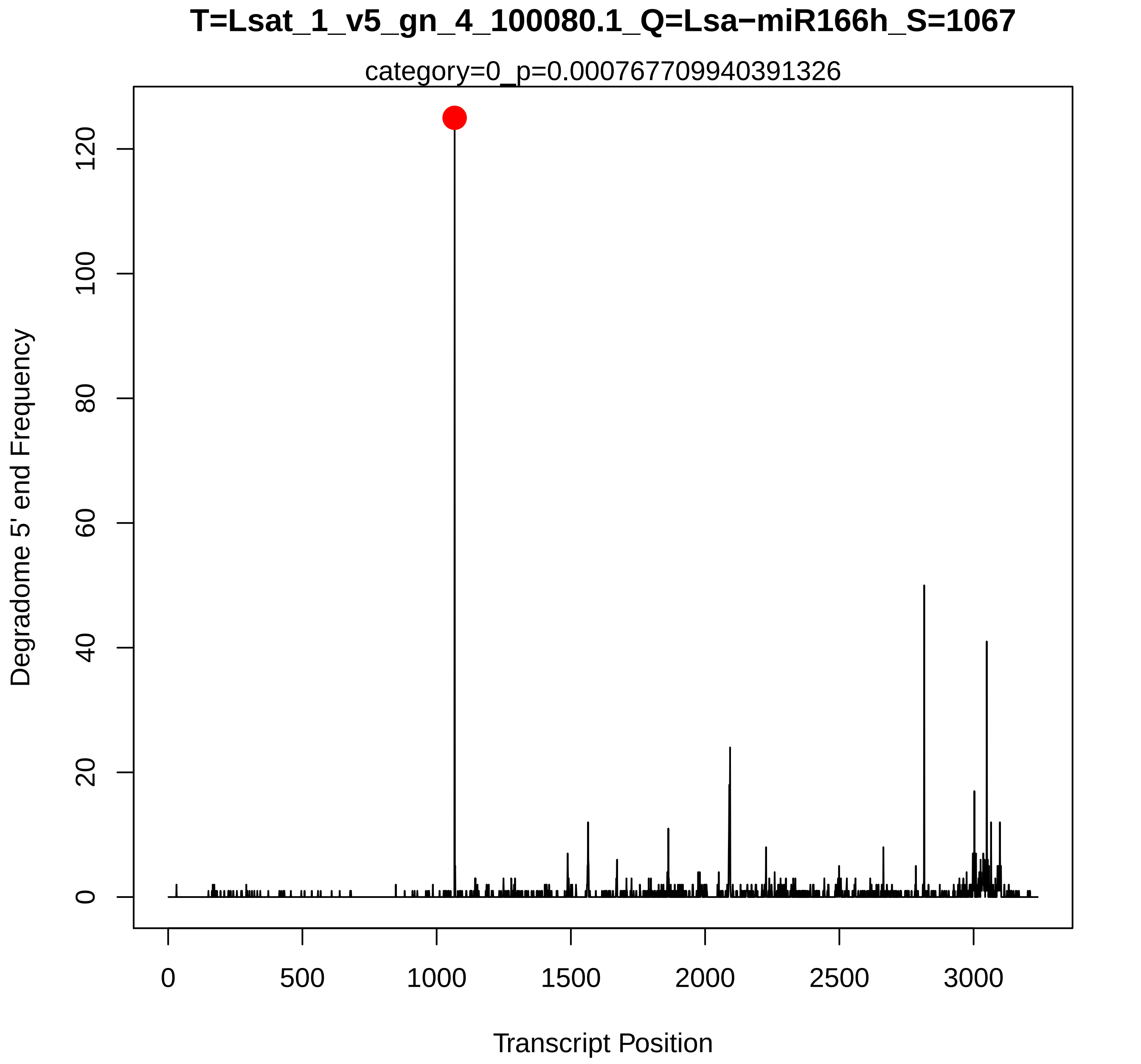
T=Lsat_1_v5_gn_4_100080.1_Q=Lsa−miR166h_S=1067
category=0_p=0.000767709940391326
Degradome 5' end Frequency
Transcript Position
0
20
40
60
80
100
120
0
500
1000
1500
2000
2500
3000
